# Physical Activity Behavior and Mental Health Among University Students During COVID-19 Lockdown

**DOI:** 10.3389/fspor.2021.682175

**Published:** 2021-07-09

**Authors:** Kathryn E. Coakley, David T. Lardier, Kelley R. Holladay, Fabiano T. Amorim, Micah N. Zuhl

**Affiliations:** ^1^Department of Individual, Family, and Community Education, College of Education and Human Sciences, University of New Mexico, Albuquerque, NM, United States; ^2^Brooks Rehabilitation College of Healthcare Sciences, Jacksonville University, Jacksonville, FL, United States; ^3^School of Health Sciences, Central Michigan University, Mt. Pleasant, MI, United States

**Keywords:** exercise, depression, sedentary behavior, COVID-19, students

## Abstract

**Background:** The coronavirus disease 2019 (COVID-19) pandemic placed social, travel, school access, and learning restrictions on University students. Excessive restriction measures have been shown to have negative impacts on mental health. Physical activity preserves mental health, and may be useful during quarantines.

**Purpose:** Explore physical activity and sedentary behavior and associations with depression and anxiety symptoms among University students during COVID-19 restrictions in the Fall 2020 semester.

**Methods:** Six hundred and ninety-seven undergraduates (18–25 years) from a U.S. public University completed a cross-sectional survey in fall 2020. The survey included demographic questions, the Generalized Anxiety Disorder Scale 7 (GAD-7), the Patient Health Questionnaire 9 (PHQ-9), and questions about meeting moderate to vigorous physical activity (MVPA) recommendations and sedentary behavior.

**Results:** Forty-nine percent did not meet MVPA guidelines. Patient Health Questionnaire 9 (*p* = 0.002) and GAD-7 (*p* = 0.024) scores were higher among those who did not achieve MVPA. Sitting time (h/day) was a significant associated with depression (*B* = 0.29 (0.06), *p* < 0.05, 95% CI = 0.18, 0.41) and anxiety (*B* = 0.24 (0.05), *p* < 0.05, 95% CI = 0.13, 0.34) severity.

**Conclusion:** Physical activity was associated with mental health among University students during COVID-19 lockdowns.

## Introduction

The Coronavirus disease 2019 (COVID-19) pandemic has led to more than 100 countries implementing various forms of restriction measures during 2020 (Dunford et al., 2020). Social distancing, travel restrictions, curfews, and school closures have been combined with hygienic efforts to slow the spread of COVID-19. Modeling studies have reported that these public health measures are important to reduce virus incidence and mortality, and may proceed into 2022 (Kissler et al., 2020; Nussbaumer-Streit et al., 2020). While effective, the social and economic impacts of lockdowns may have negative consequences for psychological health. Excessive quarantining is linked to symptoms of depression, anxiety, irritability, and insomnia (Brooks et al., 2020). Factors such as boredom, misinformation, supply concerns, frustration, and financial loss are stressors that trigger a change in mood state and may also lead to a decline in mental health (Brooks et al., 2020). Survey results among individuals from pre and during the pandemic reported a three-fold increase in depression, and those with lower social and economic resources had higher levels of depression (Ettman et al., 2020). These are all correlated with increased suicidal ideations, particularly amid Covid-19 lockdowns (Chen et al., 2020).

Of particular concern are University and college students, who have been faced with the stress of remote learning and rapid life adjustments due to COVID-19-related restrictions (Husky et al., 2020; Sahu, 2020). A small survey of 195 University students noted increased stress and anxiety among 71% of the participants (Son et al., 2020). Further, the Centers for Disease Control and Prevention (CDC) reported 63% of college-aged young adults (18–24 years of age) in the U.S. had elevated depression or anxiety between May and June 2020 (Czeisler et al., 2020). Elevated mental stress among young people may result from inadequate coping skills in the face of COVID-19-related social isolation and boredom; and, even more exacerbated among University students who may have been displaced from the traditional campus setting, leading to learning challenges and grade insecurities (Son et al., 2020; Torales et al., 2020).

Physical activity is often overlooked as a lifestyle habit to preserve mental health. Individuals who are regularly active are less likely to be diagnosed with depression or anxiety (Goodwin, 2003). Moreover, those who met moderate to vigorous physical activity (MVPA) guidelines (defined as either 150 min/week of moderate intensity exercise or 75 min/week of vigorous intensity exercise; or, an equivalent combination of both, by the United States Health and Human Services and World Health Organization) reported a 39% reduction in monthly days of poor mental health (Piercy et al., 2018; Bull et al., 2020). Individuals who were active but did not meet guidelines demonstrated a 25% reduction in days of poor mental health as well, indicating even a small amount of exercise is beneficial (Piercy et al., 2018; Fluetsch et al., 2019). In light of these findings, the World Health Organization (WHO) recently stated that adults should limit the amount of sedentary time and replace it with physical activity of any intensity or type (Bull et al., 2020; Dempsey et al., 2020). These data suggest that University students who consistently participate in physical activity may be less likely to experience depression and anxiety related to COVID-19 restrictions. Unfortunately, evidence has demonstrated a high prevalence of sedentary behavior among University students (Arias-Palencia et al., 2015). This may inadvertently influence mental health among this group (Lee and Kim, 2019).

Therefore, the aim of this study was to explore physical activity and sedentary behavior associations with depression and anxiety symptoms among young adult University students during COVID-19 restrictions in the Fall 2020 semester.

## Methods

### Recruitment

Undergraduate students 18–25 years of age at a large public University in the southwest region of the U.S were recruited for the observational cross-sectional study. Participants were enrolled at the University in spring 2020, at the start of the COVID-19 pandemic, and in fall 2020, during a second wave of cases. Four thousand students were contacted via email during a 2-week period in October–November 2020. Selection was random to create a representative sample of the University population by gender, race/ethnicity, and campus enrollment. At the end of the survey, participants could provide their email address to receive one of twenty US$50 gift card incentives. Consent was obtained when the survey was started; no consent signature was required. The study was approved by the University's Institutional Review Board (protocol 1654391). The STROBE statement was utilized as a guide to report results (see Supplemental File).

### Data Collection

Data were collected via Opinio, an online survey tool approved for research. The survey included screening questions assessing age and student status (current student and student in spring 2020). The survey then included demographic questions (age, race/ethnicity, gender, campus enrollment, academic standing, employment). The seven-item Generalized Anxiety Disorder Scale-7 (GAD-7), a validated measure of anxiety symptoms in the past 2 weeks, was used to assess anxiety within the survey (Löwe et al., 2008). All seven questions were administered and the 3-point scoring system was used to assess anxiety (0–3 for each question). The Patient Health Questionnaire-9 (PHQ-9) a validated measure of depressive symptoms in the past 2 weeks was also administered (Kroenke et al., 2001). Nine questions were asked and a 0- to 3-point scale was used to score the outcome. For both the GAD-7 and PHQ-9 questionnaires, the scores for each individual could not be validated by a clinician due to the survey nature of the study (Kroenke et al., 2001). Physical activity measures included moderate intensity exercise minutes per week; vigorous intensity exercise minutes per week; walking days per week; and number sitting hours per day. Questions examining moderate and vigorous intensity exercise were based on the International Physical Activity Questionnaire (IPAQ), which is validated for young adults (Craig et al., 2003). Specifically, only Part 4 of the IPAQ was used, which assesses recreation, sport, and leisure -time physical activity over the previous seven days (Craig et al., 2003).

Those who reported 150 min or more of moderate intensity exercise per week (≥150 min/week) and, or 75 min or more of vigorous intensity exercise per week (≥75 min/week), or an equivalent combination of both moderate- and vigorous-intensity aerobic exercise were considered to meet physical activity guidelines (USDHHS, 2018). Combination was calculated by adding moderate minutes per week plus two times vigorous minutes per week. Meeting MVPA was determined if combination equated to ≥150. Number of hours sitting per day was examined as a continuous variable (0–24).

### Data Analysis

*A-priori* power analyses were conducted in G^*^Power (Erdfelder et al., 1996) to determine adequate sample size to identify medium to large effect sizes based on Cohen's criteria (Cohen, 1988). Power analyses indicated that with a desired power of 0.80, two-tailed, alpha = 0.05, medium effect size of 0.30, a sample size >100 was adequate to identify significant effects cross-sectionally. Power estimates ranged from 0.65 (*d* = 0.24) and 0.95 (*d* = 0.38). Only those who completed the physical activity portion of the survey were included in the analyses. Baseline demographic data are presented descriptively. Those who reported meeting moderate intensity guidelines (≥150 min/week) or vigorous intensity guidelines (≥75 min/week), or an equivalent combination of both moderate and vigorous intensity exercise were considered to meet MVPA recommendations. Those that did not achieve either moderate or vigorous intensity guidelines were categorized into not meeting MVPA. The GAD-7 and PHQ-9 were scored according to standard guidelines. Generalized Anxiety Disorder Scale 7 scores range from 0 to 21 and were used to categorize anxiety symptoms severity: mild (5–9), moderate (10–14), or severe (>15). Patient Health Questionnaire 9 scores range from 0 to 27 and were used to categorize depression symptom severity: mild (5–9), moderate (10–14), moderately severe (15–19), or severe (>20). Age, GAD-7 score, PHQ-9 score, minutes of exercise, and sitting time were compared between groups (meeting MVPA recommendations vs. not meeting MVPA recommendations) using Mann-Whitney U-tests. The relationships between GAD-7 and PHQ-9 scores and physical activity intensity (moderate and vigorous min/week) and sitting time (h/day) were analyzed using Spearman rank-order correlation. Last, multivariate linear regression analyses examined associations between exercise intensity (including both moderate and vigorous intensity min/week), walking time (min/week), and sitting times and PHQ-9 score indicating depression severity and GAD-7 score indicating anxiety severity, adjusted for sociodemographic covariates. Covariates were retained based on meaningful contribution and statistical significance to the final analytical model (Aneshensel, 2012). Gender was the only covariate that contributed significantly. Both age and employment status were evaluated but did not qualify.

## Results

The survey was distributed via email to 4,000 randomly selected students in late October 2020. A total of 852 started the survey, 75 were removed due to not meeting exclusion criteria designating a sample of 777 participants in the parent study (Coakley et al., 2021). For the current study, another 80 did not complete the physical activity section of the survey and were removed from subsequent analyses. Therefore, 697 undergraduates 18–25 years of age were included. Average age was 21.29 ± 1.62 years. Sixty-two percent (*n* = 431) were female; 31.6% (*n* = 220) were White; 29% were Hispanic, Latino, or Spanish (*n* = 204); and 22.7% (*n* = 158) reported being multiracial. Of note, in Fall 2020 the undergraduate population was 57% female; 30% white, 50% Hispanic, 3% African American, and 4% were multiracial. The majority of survey responders (47.2%, *n* = 329) were employed part-time, and 35% were not employed (see Table 1). No missing data were present among main analytic variables including GAD-7 (measure of anxiety) and PHQ-9 (measure of depression).

**Table 1:** Demographic characteristics of study participants.

**Demographics**	***n***	**%**
**Mean age (years)** **=** **21.29** **±** **1.62**	697	100
**Gender**		
Male	238	34
Female	431	62
Transgender, gender fluid, other	28	4
**Race/Ethnicity**		
American Indian/Alaskan Native	53	7.6
Asian	54	7.7
Black or African American	8	1.1
Hispanic, Latino, or Spanish	204	29.3
White	220	31.6
Multiracial	158	22.7
**Employment**		
Employed, full-time	98	14.1
Employed, part-time	329	47.2
Not employed, looking for work	128	18.4
Not employed, not looking for work	120	17.2
Other	22	3.2
**Mental health**		
GAD-7 overall mean = 10.49 ± 6.07		
Mild (score 5–9)	187	27
Moderate (score 10–14)	173	25
Severe (score ≥15)	145	21
PHQ-9 overall mean = 11.25 ± 6.92		
Mild (score 5–9)	150	22
Moderate (score 10–14)	172	25
Moderately severe (score 15–19)	144	21
Severe (score ≥ 20)	89	13
**Physical activity**	**Mean** **±*****SD***	
Moderate intensity exercise (min/week)	87.79 ± 136.23	
Vigorous intensity exercise (min/week)	94.71 ± 123.09	
Walking days (days/week)	3.88 ± 2.52	
Sitting time (h/day)	8.61 ± 4.38	

*GAD-7, Generalized Anxiety Disorder Scale 7; PHQ-9, Patient Health Questionnaire 9*.

### Physical Activity Exercise Recommendations

A total of 350 participants (51%) achieved either ≥150 min/week of moderate intensity or ≥75 min/week aerobic exercise, or combination of both (Table 2). Those who met MVPA guidelines, reported a total of 334.22 ± 213.98 min/week of aerobic activity compared to 28.26 ± 36.28 min/week among those who did not meet MVPA guidelines (*p* < 0.0001). Both moderate intensity exercise minutes (156.48 ± 162.98 vs. 18.27 ± 31.80 min/week, *p* < 0.0001) and vigorous intensity exercise minutes (177.80 ± 126.23 vs. 9.98 ± 17.21 min/week, *p* < 0.0001) were higher among individuals who met MVPA guidelines. Those who did not meet MVPA guidelines reported significantly higher sitting time per day (*p* < 0.0001), along with PHQ-9 (*p* = 0.004) and GAD-7 (*p* = 0.024) scores.

**Table 2:** Students who reportedly met physical activity weekly MVPA recommendations (reported as either moderate, ≥ 150 min/week); and, or vigorous, ≥ 75 min/week) compared to those that did not meet guidelines.

**Outcome**	**Meeting MVPA recommendations**	**Not meeting MVPA recommendations**	***p*-Value**
Sample (*n*)	350	347	
Age	21.24 ± 1.58	21.33 ± 1.67	
Total minutes of moderate-vigorous exercise/week ^τ^	334.22 ± 213.98^*^	28.26 ± 36.28	<0.0001
Minutes of moderate exercise/week	156.48 ± 162.98^*^	18.27 ± 31.80	<0.0001
Minutes of vigorous exercise/week	177.80 ± 126.23^*^	9.98 ± 17.21	<0.0001
PHQ score	10.49 ± 6.85	12.10 ± 6.94^*^	0.002
GAD score	10.00 ± 5.97	11.04 ± 6.14^*^	0.024
Sitting time per day (h)	7.84 ± 3.87	9.41 ± 4.71^*^	<0.0001

*MVPA, moderate-vigorous physical activity; GAD-7, Generalized Anxiety Disorder Scale 7; PHQ-9, Patient Health Questionnaire 9*.

* *Significantly different, p < 0.05*.

τ *Summation of moderate and vigorous exercise minutes per week*.

### Physical Activity and Mental Health

Generalized Anxiety Disorder Scale 7 scores were positively correlated with sitting time (*r*_*s*_ = 0.17, *p* < 0.0001), and had a negative association with vigorous exercise min/week (*r*_*s*_ = 0.082, *p* = 0.03). Similarly, PHQ-9 were correlated with sitting time (*r*_*s*_ = 0.19, *p* < 0.0001); and negatively correlated with moderate exercise min/week (*r*_*s*_ = −0.080, *p* = 0.03); and with vigorous exercise min/week (*r*_*s*_ = −0.10, *p* = 0.007).

### Multivariate Linear Regression Analyses

Multivariate regression analyses for PHQ-9 score indicating depression symptom severity are presented in Table 3. Results indicated that sitting time per day (h) was positively associated with depression symptoms [*B* = 0.29 (0.06), *p* < 0.05, 95% CI = 0.17, 0.41]; and vigorous intensity exercise (min/week) and depression symptoms had a negative association that trended near significance [*B* = −0.28 (0.15), *p* = 0.054, 95% CI = −0.56, −0.01]. Gender was included as a covariate. Female participants displayed a positive association with depression symptom severity [*B* = 2.21 (0.48), *p* < 0.05, 95% CI = 1.27, 3.15].

**Table 3:** Multivariate regression analyses between exercise intensity, walking days, sitting times, and depression severity (based on PHQ-9 score).

	**B (SE)**	***β***	**95% CI**	
			**LL**	**UL**
Vigorous exercise (min/week)	−0.28 (0.15)^+^	−0.07	−0.56	−0.01
Moderate exercise (min/week)	0.00 (0.00)	0.02	−0.01	0.02
Walking days (days/week)	−0.03 (0.11)	−0.01	−0.25	0.18
Sitting time per day (h)	0.29 (0.06)^*^	0.19	0.18	0.41
Gender (Male = 1)	2.21 (0.48)^*^	0.17	1.27	3.15
*F*_(5, 692)_ = 11.52^**^				
*R*^2^ = 0.07				

*PHQ-9, Patient Health Questionnaire 9*.

* *Significantly different, p < 0.05*.

** *Significantly different, p < 0.001*.

+ *p-value=0.054*.

Multivariate regression analyses for GAD-7 score indicating anxiety symptom severity are presented in Table 4. Results indicated that sitting time per day (h) was positively associated with anxiety (GAD-7 score) severity [*B* = 0.24 (0.05), *p* < 0.05, 95% CI = 0.13, 0.34]. Gender was included as a covariate. Female participants showed a positive association with anxiety symptom severity [*B* = 2.25 (0.42), *p* < 0.05, 95% CI = 1.43, 3.07].

**Table 4:** Multivariate regression analyses between exercise intensity, walking intensity, sitting times, and anxiety severity (based on GAD-7 score).

	***B* (*SE*)**	***β***	**95% CI**	
			**LL**	**UL**
Vigorous exercise (min/week)	−0.14 (0.13)	−0.04	−0.39	0.11
Moderate exercise (min/week)	−0.00 (0.01)	−0.00	−0.01	0.01
Walking days (days/week)	0.10 (0.09)	0.04	−0.09	0.29
Sitting time per day (h)	0.24 (0.05)^*^	0.17	0.13	0.34
Gender (Male = 1)	2.25 (0.42)^*^	0.20	1.43	3.07
F_(5, 692)_ = 11.18^**^				
*R*^2^ = 0.08				

*GAD-7, Generalized Anxiety Disorder Scale 7*.

* *Significantly different, p < 0.05*.

** *Significantly different, p < 0.001*.

## Discussion

A physically active lifestyle supports mental health (Goodwin, 2003). This study identified that University students that did not achieve recommended MVPA weekly targets had higher PHQ-9 and GAD-7 scores indicating more severe symptoms of depression and anxiety, respectively. In addition, through multivariate regression analysis, amount of daily sitting time had a significant association with both more severe depression and anxiety symptoms during the COVID-19 restrictions among University students in fall 2020.

In 2018, the U.S Department of Health and Human Services, along with the WHO in 2020 updated physical activity guidelines (Piercy et al., 2018; USDHHS, 2018). The recommendations suggest that adults should aim to achieve at least 150–300 min of moderate intensity aerobic exercise, or 75–150 min of vigorous intensity aerobic exercise per week, or an equivalent of both. For the first time, the new guidelines highlight the benefits of physical activity for several conditions affecting the brain, such as depression, anxiety, and Alzheimer's disease; however, nearly 80% of Americans do not meet guidelines. Data from this study demonstrated that 49% of University student participants did not meet MVPA exercise goals. Those who reported less than MVPA targets, determined by evaluating weekly moderate intensity (150 min/week) and vigorous intensity (75 min/week) exercise behavior, scored higher on the PHQ-9 for depression and GAD-7 for anxiety, compared to those who achieved the MVPA targets. Correlation analysis also revealed that PHQ-9 scores were negatively associated moderate exercise minutes per week, and trended with weekly vigorous intensity exercise. Accordingly, individuals with more severe symptoms of depression and anxiety reported lower moderate and, or, vigorous exercise minutes per week. These data suggest that University students who achieve PA recommendations during COVID-19 restrictions were likely to have a lower depression and anxiety symptoms. However, reverse causality cannot be ruled out based on these data. It is possible that elevated depression related to COVID-19 lockdown measures contributed to lack of physical activity. While less studied, data has suggested a link between depression and development of a sedentary lifestyle (Roshanaei-Moghaddam et al., 2009). Specifically, individuals with elevated baseline depressive symptoms were 1.79 times more likely to be physically inactive (Allan et al., 2007). In addition, young people with mood disorders are at increased risk of developing poor health behaviors such as smoking and excessive weight gain (Katon, 2003; McElroy et al., 2004). Young adults with symptoms such as low energy levels, irritability, anxiousness, and lack of self-esteem, along with co-existing negative health habits would likely lack motivation to exercise, nor see the mental health-related benefits of being physically active.

Several observational studies have demonstrated a positive impact of PA on symptoms of depression among women, minority populations, men, and University students (Wise et al., 2006; McKercher et al., 2009; Currier et al., 2020; Tao et al., 2020). Of note, the majority of survey responders in the current study were female (62%), and of minority ethnic backgrounds (~66%). These findings suggest that physical activity may protect against mental health decline in young adults during COVID-19 lockdowns. Limited comparative data exists regarding the role of PA on symptoms of depression during the COVID-19 pandemic among young people. Pieh et al., reported a higher prevalence of depression (PHQ-9 > 10) among adult Austrians, and particularly young adults (<35 years of age) after 4-weeks of lockdowns, and a nearly five-fold increase compared to pre-pandemic times (Pieh et al., 2020).

To date, there have been no accurate evaluations of PA and depressive symptoms during the COVID-19 pandemic among only young adults. In Spain, it was demonstrated that those who met PA guidelines for moderate or vigorous exercise had lower depression scores during COVID-19 lockdowns, but only older adults 60–92 years of age were included (Carriedo et al., 2020). Similar relationships between PA and depressive symptoms were reported in Norway among 1,281 adults, aged 19–90 years (Ernstsen and Havnen, 2020). Early pandemic data from spring and summer 2020 have suggested an alarming mental health decline, notably among young adults, which may be due to a lack of developed coping and emotional behaviors (Compas et al., 2014; Czeisler et al., 2020). In addition, young people are less likely to recognize their mood/behavior changes, and seek therapeutic assistance (Pedersen and Paves, 2014). The current data support the notion that young University students who exercise more and achieve PA recommendations are less likely to experience symptoms of depression. Therefore, encouraging PA behavior may be a therapeutic option. However, the causal effect cannot be evaluated.

Symptoms of anxiety were higher among those that did not meet MVPA recommendations, and anxiety symptoms severity was inversely associated with weekly minutes of vigorous exercise. A similar relationship between physical activity and perceived anxiety was observed among 2,250 middle-aged adults during the Fall 2020 COVID-19 lockdown in Spain (López-Bueno et al., 2020). Those who met WHO physical activity recommendations were nearly half as likely to report elevated anxiety (López-Bueno et al., 2020). Meta analytical findings have demonstrated a small reductive effect of physical activity on symptoms of anxiety among non-clinical populations (Rebar et al., 2015). Mechanisms include psychological changes such as mood enhancement and self-efficacy, along with neurophysiological adaptations (Portugal et al., 2013). However, conflicting data regarding the benefits of exercise on symptoms of anxiety have been reported (Bartley et al., 2013). The multivariate analyses in the current study revealed a non-association between physical activity (both moderate and vigorous intensity) and symptoms of anxiety. One explanation is that the anxiolytic effect of exercise is transient, termed the “endorphin effect,” and is diminished after a short period of time (Anderson and Shivakumar, 2013). In addition, the impact of exercise training may be more evident among those who have clinically diagnosed anxiety (Herring et al., 2010).

Sitting time (h/week) was positively associated of depression symptom severity and vigorous intensity exercise had a negative association that was trending toward significance (*p* = 0.054). Female gender identity was also associated with depression severity. Sitting time and female gender identity were also significantly associated with anxiety symptom severity. The average sitting time per day for this cohort of University students was 8.61 h/day, which is similar to the 7.59 h/day reported in a recent survey among 244 students (Lee and Kim, 2019). Activities performed while sitting were not evaluated in the current study, but recent data suggest that passive behaviors such as watching television worsened depressive symptoms, and television watching time has surged since the COVID-19 pandemic (Dixit et al., 2020; Huang et al., 2020). Among University students, an increase in sitting time has been linked to both anxiety and depression (Lee and Kim, 2019). Moreover, due to COVID-19 restrictions, physical activity may have been replaced by sedentary behavior and sleep among young adults (Zheng et al., 2020). The current data reveals that every additional hour of sitting time was associated with an increase in PHQ-9 score by 0.19, and GAD-7 score by 0.17 while holding other predictors constant. Interestingly, in a study published in 2001, low levels of physical activity such as achieving at least 75 min per week of any type of PA was associated with 45% lower prevalence of depression among women (Dunn et al., 2001). This equates to ~10 min per day of activity and could be accomplished by simply walking outside. Therefore, fully achieving PA recommendations may not be required to prevent an increase in symptoms of depression and anxiety; however, avoiding sitting time is very important for mental health (Dempsey et al., 2020).

The results of the study should be interpreted with several limitations in mind. First, the response rate was lower (17%) than desired for an online survey; however, the sample size is comparable to similar survey studies that analyzed PA and mental health during COVID-19 lockdowns (Nulty, 2008; Barkley et al., 2020; Carriedo et al., 2020). Second, the survey was conducted at one University in the Southwest region of the U.S., and may not be generalizable to all undergraduate students. Third, survey completers may not have understood the difference between moderate and vigorous exercise, which may have influence reported data. Fourth, the IPAQ was used to assess moderate and vigorous exercise, and groups were allocated based on their physical activity. The IPAQ is reported to be reliable among young adults, but has also been reported to overestimate PA (Papathanasiou et al., 2009; Lee et al., 2011). Fifth, the PA and sedentary behavior was assessed by self-report, which is potentially subject to misreporting. However, this study used a short-term recall of PA activity (last 2 weeks) which may reduce the magnitude of reporting errors (Matthews et al., 2012). Lastly, no previous or current diagnoses or treatment of depression and anxiety was assessed.

## Conclusion

It is clear that COVID-19 restrictions and the prolonged nature of a global pandemic are linked to a decline in mental health among young adult University students (Czeisler et al., 2020; Son et al., 2020). Social isolation, campus displacement, and academic-related stress (assignments and online transitions) may be linked to elevations in symptoms of depression and anxiety. Physical activity is an important stress relieving behavior, and students that achieved recommended MVPA guidelines had lower levels of depression, but the majority of University students do not meet PA recommendations. Moreover, sedentary behavior (evaluated as sitting time per day) was significantly associated with both depression and anxiety symptom severity. Efforts should be made to encourage students to engage in some form of PA each day, and to avoid sitting time. As little as 10 min per day of exercise has been shown to support mental health, and the implementation of home-based exercise training protocols such as bodyweight exercises may be a simple strategy to reducing symptoms of depression and anxiety (Rebar and Taylor, 2017).

## Data Availability Statement

The raw data supporting the conclusions of this article will be made available by the authors, without undue reservation.

## Ethics Statement

The studies involving human participants were reviewed and approved by University of New Mexico IRB. The patients/participants provided their written informed consent to participate in this study.

## Author Contributions

KC conceived the study and carried out methodology and data collection. DL performed data collection and data analyses. KH and FA assisted with methodology, data collection, and analyses. MZ contributed to methodology and data analyses and also complete the first draft of manuscript. All authors contributed to manuscript revisions, and table/figure development.

## Conflict of Interest

The authors declare that the research was conducted in the absence of any commercial or financial relationships that could be construed as a potential conflict of interest.
